# *In vivo* H1 MR spectroscopy using 3 Tesla to investigate the metabolic profiles of joint fluids in different types of knee diseases

**DOI:** 10.1120/jacmp.v17i2.6144

**Published:** 2016-03-08

**Authors:** Wook Jin, Dong‐Cheol Woo, Geon‐Ho Jahng

**Affiliations:** ^1^ Department of Radiology Kyung Hee University Hospital at Gangdong College of Medicine Kyung Hee University Seoul 134‐727 South Korea; ^2^ Biomedical Research Center Asan Institute for Life Sciences Asan Medical Center University of Ulsan College of Medicine Seoul 138‐736 South Korea

**Keywords:** *in vivo* proton spectroscopy, magnetic resonance, effusion, knee, lipid metabolites

## Abstract

*In vivo* proton (H1) magnetic resonance spectroscopy (MRS) has not yet been systematically used to study joint fluids in human knees. The objective of this study, therefore, was to assess the ability of proton MRS to identify the apparent heterogeneous characteristics of metabolic spectra in the joint fluid regions in human knees using a high‐field MRI system. Eighty‐four patients with effusion lesions who were referred for routine knee MR imaging underwent proton MRS with point‐resolved, single‐voxel MR spectroscopy using a clinical 3.0 Tesla MRI system. Thirty‐eight patients were confirmed to have the following: degenerative osteoarthritis, 21 patients (Group 1); traumatic diseases, 12 patients (Group 2); infectious diseases, 4 patients and an inflammatory disease, 1 patient (Group 3). Spectroscopy data were analyzed using the public jMRUI freeware software to obtain lipid metabolites. Nonparametric statistical comparisons were performed to investigate any differences in metabolites among the three disease groups. The major metabolites were vinylic CH=CH lipids around 5.1−5.5 ppm, ${\text{CH}}_{2}$ lipids around 1.1−1.5 ppm, and ${\text{CH}}_{3}$ lipids around 0.7−1.0 ppm. Each patient had either a CH=CH lipid peak, ${\text{CH}}_{2}$ and ${\text{CH}}_{3}$ lipid peaks, or all three peaks. There were no significant differences among the three groups for the ${\text{CH}}_{3}$ (p=0.9019), ${\text{CH}}_{2}$ (p=0.6406), and CH=CH lipids (p=0.5467) and water (p=0.2853); none of the metabolites could differentiate between any of the three types of diseases. The ${\text{CH}}_{2}$ lipids in the 38 patients who had confirmed fluid characteristics were significantly correlated with ${\text{CH}}_{3}$ lipids (rho=0.835, p<0.0001). The ratio of ${\text{CH}}_{3}$ to ${\text{CH}}_{2}$ was highest in the degenerative disease. In both the degenerative and traumatic diseases, metabolite peaks of the vinylic CH=CH lipids around 5.1−5.5 ppm and of the sum of the ${\text{CH}}_{2}$ and ${\text{CH}}_{3}$ lipids around 0.7−1.5 ppm were observed, but in the infectious disease, only a metabolite peak of the sum of the ${\text{CH}}_{2}$ and ${\text{CH}}_{3}$ lipids was detected. Although none of the metabolites could statistically significantly differentiate between the three types of diseases, the different lipid metabolite peaks and their ratios in the three disease groups may give us a hint at the different mechanisms of joint fluids in the infectious, degenerative, and traumatic diseases.

PACS number(s): 87.61.Ff, 33.25.+k, 87.14.Cc

## I. INTRODUCTION

Knee effusion occurs when excess fluid accumulates in the knee. Knee joint fluid may consist of hemorrhage, hypertrophic synovium, and exudation from inflamed tissues. Effusions that arise in patients with degenerative arthritis are viscous, but those that arise in patients with inflamed synovia are aqueous.^1^, ^2^ The fluid is composed of a number of metabolites, especially lipids,^(3)^ and shows high signal intensity on T2‐weighted or intermediated (or proton)‐weighted images and low signal intensity on T1‐weighted images.^(4)^ During a disease's progression, a metabolic component is released from the cartilage matrix into the joint fluid. Therefore, joint effusion analysis should yield important information for understanding disease progressions and characterizations. Noninvasive characterizations of effusion regions on the basis of imaging may obviate the need for joint aspiration with its associated patient morbidity.

Proton (H1) MR spectroscopy (MRS), a noninvasive method to identify metabolic components in regions of interest, provides information on molecular characterizations, and variations in metabolite signals could represent different pathologic conditions.^5^, ^6^, ^7^, ^8^, ^9^ Proton MRS has been used extensively to investigate tumor metabolism in brain lesions, but its use has been limited in musculoskeletal lesions.^9^, ^10^, ^11^, ^12^ Recent studies that used proton MRS have opened up the feasibility of investigating muscle metabolism.^7^, ^13^, ^14^ Proton MRS studies have been performed to evaluate musculoskeletal tumors^8^, ^9^, ^12^, ^15^, ^16^ and muscle metabolism in neuromuscular diseases^(7)^ and to study human muscle with normal and different pathological conditions.^5^, ^6^, ^13^, ^14^, ^17^

A number of studies have used *ex vivo* nuclear magnetic resonance (NMR) spectroscopy on synovial fluid samples to investigate their metabolic profiles,^3^, ^18^, ^19^, ^20^, ^21^ and a wide variety of low‐molecular‐mass components with ${\text{CH}}_{2}$ and ${\text{CH}}_{3}$ lipid peaks were identified in the samples.^18^, ^22^, ^23^, ^24^ In musculoskeletal lesions, the major metabolites are included in lipids with other metabolites. Lipids are major substrates for energy production both at rest and during muscle contraction, and they may flow from the muscle to the joint space. Therefore, measurements of lipid peak resonance signals in *in vivo* MR spectroscopy may be reliable for obtaining information on effusion mechanisms to understand disease progressions.^20^, ^22^, ^23^, ^25^

Although some previous *ex vivo* NMR studies reported the possibility of monitoring pathologic conditions in fluid‐filled lesions,^3^, ^22^ *in vivo* proton MRS has not yet been systematically used to study the joint fluids in human knees. The objective of this study, therefore, was to assess the ability of proton MR spectroscopy to identify the apparent heterogeneous characteristics of metabolic spectra in effusion regions in human knees using a high‐field MRI system.

## II. MATERIALS AND METHODS

### A. Patient populations

This study was performed with the approval of the local institutional review board. Eighty‐four patients (mean age=45 yrs, standard deviation (SD)=16 yrs; range=7−81 yrs, 43 males and 41 females) with effusion lesions who had been referred for routine knee MRI underwent point‐resolved spectroscopy (PRESS) single‐voxel proton MRS using a clinical 3.0 Tesla MRI system (Philips, Achieva, Best, The Netherlands) with a dedicated 8‐channel knee coil. In 38 of the 84 patients, the causes of fluid accumulations were confirmed using clinical data, joint fluid analysis, or pathology reports as follows: 21 patients had degenerative osteoarthritis (Group 1), 12 patients had traumatic diseases (Group 2), and 4 patients and 1 patient had, respectively, infectious diseases and an inflammatory disease (Group 3). We did not have final diagnostic results for the other 46 patients. Patient characteristics are listed in Table 1.

**Table 1:** Summary of the demographic characteristics and average intensities of lipid metabolites and water for the three different disease groups

	*Differential Diagnosis (DDX)*				*Unknown DDX*
	*Degenerative*	*Traumatic*	*Infectious and Inflammatory*	*p‐value*	*Unknown*
Patient Number (84)	21	12	5		46
Age (ys)	59±8.746	36±15.06	43.6±22.65	0.0007	41.78±13.97
Gender (Male/Female)	3/18	10/2	3/2	0.0004	27/19
*Metabolite Intensities (p‐value, Kruskal‐Wallis test)*					
${\text{CH}}_{3}$ lipid (0.7−1.1 ppm)	0.0026±0.0045	0.0018±0.0022	0.0016±0.0013	0.9019	0.0017±0.0038
${\text{CH}}_{2}$ lipid (1.1−1.5 ppm)	0.0108±0.0153	0.0089±0.0098	0.0102±0.0062	0.6406	0.0146±0.0326
CH=CH lipid (5.1−5.5 ppm)	0.0035±0.0039	0.0046±0.0056	0.0011±0.0011	0.5467	0.0042±0.0043
Water (4.7 ppm)	0.7415±0.9041	0.5037±0.2534	0.3340±0.1583	0.2853	0.6337±0.4554
*Ratios Among Metabolites (p‐value, Kruskal‐Wallis test)*					
Both ${\text{CH}}_{3}$ and ${\text{CH}}_{2}$ lipids	0.0134±0.0198	0.0108±0.0117	0.0118±0.0074	0.6886	0.0163±0.0351
Ratio ${\text{CH}}_{3}/{\text{CH}}_{2}$	0.3012±0.2079	0.2190±0.1703	0.1554±0.0464	0.1700	0.2938±0.2642
Ratio $\left(\text{CH}=\text{CH})/({\text{CH}}_{2}+{\text{CH}}_{3}\right)$	0.9922±1.7110	0.9153±1.2953	0.1431±0.1368	0.3696	1.5772±2.6631
LI	0.3793±0.5232	0.3810±0.5898	0.7677±0.1901	0.3696	0.2537±0.6233
Number of Patients in Each Spectrum Type					
Type A (%)	13 (61.9%)	7(58.3%)	5(100%)	0.5500	23(50%)
Type B (%)	5(23.8%)	3(25.0%)	0(0%)		19(41.3%)
Type C (%)	3(14.3%)	2(16.7%)	0(0%)		4(8.7%)

Data show mean ± SD (arbitrary unit).

Age was significantly different among the groups (Kruskal‐Wallis test).

Gender was significantly different among the groups (Chi‐squared test).

Spectrum types were not different among the three groups (Chi‐squared test).

LI=lateralization index, calculated as LI=(PA−PB)/(PA+PB), where PA was the sum of the peak intensity of ${\text{CH}}_{2}$ and ${\text{CH}}_{3}$ and PB was the sum of CH=CH; Type A (0.7−1.5 ppm)=MRS spectrum that showed dominant peaks for both ${\text{CH}}_{2}$ and ${\text{CH}}_{3}\;\text{lipids}$; Type B (5.1−5.5 ppm)=MRS spectrum that showed a dominant peak for the CH=CH lipid; Type C=MRS spectrum that showed both Type A and B metabolite peaks.

### B. Spectrum acquisitions

**Figure 1:**
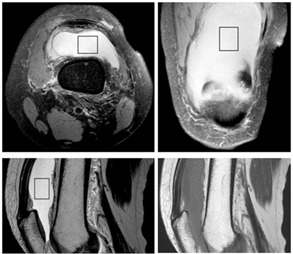
A defined MR spectroscopic voxel at an effusion area in a knee on transverse and coronal proton density‐weighted, fat‐saturated images and sagittal T2‐weighted and T1‐weighted images in a patient (48‐year‐old, female).

The single‐voxel, point‐resolved spectroscopy (PRESS) sequence was added to the routine knee MRI protocol and was performed before contrast administration to avoid possible changes in spectrum baselines caused by contrast material in patients who required a contrast medium. The PRESS MRS images were acquired with water suppression accomplished with chemical shift selective sequence (CHESS) pulses with a bandwidth of 160 Hz, applied on‐resonance with the water signal. The spectra of unsuppressed water signals were also acquired in order to take advantage of the water peaks for relative quantification analysis. Scan conditions were: TR=2000 ms, TE=40 ms, averages=128, spectral bandwidth=2000 Hz, and sampling points=2048; the scan time was 4.5 min. The spectral data were obtained from varying voxel sizes depending on the sizes of the effusion areas, but the minimal voxel size was limited to $1\;{\text{cm}}^{3}\;(10\;\text{mm}\times 10\;\text{mm}\times 10\;\text{mm}$) localized to the insides of the effusion areas. The location of spectral volume was placed within the central portion of the joint fluid area to be fitted to the size of the effusion area as well as to be excluded in the synovium, which is usually located at the peripheral portion of the joint fluid. In order to localize the voxel location of the spectroscopy, transverse, coronal, and sagittal images were obtained using a T2‐weighted turbo spin‐echo sequence. Figure 1 shows a defined MR spectroscopic voxel at the effusion area in the knee on transverse and coronal proton density‐weighted, fat‐saturated images and sagittal T2‐weighted and T1‐weighted images in one patient (48‐year‐old, female). We usually used proton density‐weighted, fat‐saturated images and T2‐weighted images to localize the region of interest of the spectrum acquisition.

### C. Spectrum analyses

Spectroscopy data were saved in a Philips format and transferred into a personal computer. The data were analyzed using public freeware software called jMRUI (Academy of Sciences of the Czech Republic, Brno, Czech Republic, http://www.jmrui.eu/support/scientific-technical-literature/). After the chemical shift of the water was set to 4.7 ppm, spectroscopic data were created by numeric integration over a range from 0 to 8 ppm. Spectra were processed with zero‐order phase correction based on the water peaks using Lorentzian apodization (3 Hz). Because water signals are not perfectly suppressed by CHESS, a Hankel‐Lanczos singular value decomposition (HLSVD) filter was applied to the spectrum‐suppressed water signals in postprocessing to subtract the residual water signals in the frequency domains. A nonlinear least square algorithm, called AMARES in the jMRUI software package, was used to fit the spectrum, using the Gaussian line shape for each metabolite's resonance and a Lorentzian line shape for the water peaks. Resonance peaks were assigned based on the analysis of both characteristic and previously published chemical shift values.^3^, ^18^, ^21^, ^22^, ^24^ The identities of the components responsible for the resonances in the proton MR spectra of the joint fluid samples were assigned by considering their characteristic chemical shift values. In this study, the metabolites of ${\text{CH}}_{3}$ (0.7~1.1 ppm), ${\text{CH}}_{2}$ (1.1~1.5 ppm), and CH=CH (5.1~5.5ppm) lipids as well as water were obtained from all patients. We grouped both ${\text{CH}}_{3}$ and ${\text{CH}}_{2}$ lipids in the low ppm range and CH=CH lipid in the high ppm range because the ${\text{CH}}_{3}$ and ${\text{CH}}_{2}$ lipids usually overlapped. If the ${\text{CH}}_{2}$ and ${\text{CH}}_{3}$ metabolites in the low ppm range were dominant, then we designated this spectrum Type A. If the CH=CH metabolite in the high ppm range was dominant, then the spectrum was Type B. Finally, if the metabolites in both the low and high ppm ranges were dominant, then the spectrum was Type C. In order to quantify the three types of spectra, we calculated the lateralization index (LI) as

(1) LI=(PA−PB)(PA+PB)

where *PA* was the sum of the peak intensity of ${\text{CH}}_{2}$ and ${\text{CH}}_{3}$ and PB was that of CH=CH. Therefore, LI values should have been between 1.0 and −1.0. LI=1.0 meant that the ${\text{CH}}_{2}$ and ${\text{CH}}_{3}$ lipids were dominant in the spectrum (Type A spectrum); LI=−1.0 meant that the CH=CH lipid was dominant (Type B spectrum); and LI=0.0 meant that both Type A and B metabolites were balanced in the spectrum (Type C spectrum).

### D. Statistical analyses

Before statistical evaluations of metabolites, normality was tested using the Kolmogorov‐Smirnov test for each metabolite. None of the metabolites had a normal distribution, and therefore, we used a nonparametric test for the following investigations. First, we used the Kruskal‐Wallis test^(26)^ to investigate any differences in metabolites among the three patient groups (degenerative, traumatic, and infectious and inflammatory diseases) and then the Mann‐Whitney test to compare metabolites between the degenerative and traumatic diseases. Second, to investigate the relationships between metabolites, we calculated the Spearman's coefficient of rank correlations (rho) from the ${\text{CH}}_{2},{\text{CH}}_{3}$, and CH=CH lipids and the sum of the ${\text{CH}}_{2}$ and ${\text{CH}}_{3}$ lipids. Finally, receiver operating characteristic (ROC) curve analysis was performed to investigate sensitivity and specificity using metabolites in the degenerative and traumatic disease groups. Statistical analysis was performed with MedCalc Statistical software Version 15.4 (MedCalc Software bvba, Ostend, Belgium; http//www.medcalc.org; 2015) and with significance set at p=0.05.

## III. RESULTS

**Figure 2:**
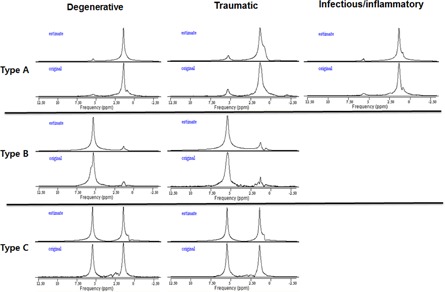
Three typical types of spectra obtained from different patients in the different disease groups. Type A is the MRS spectrum that shows dominant peaks for the ${\text{CH}}_{2}$ and ${\text{CH}}_{3}$ lipids. Type B is the MRS spectrum that shows a dominant peak for the CH=CH lipids. Type C is the MRS spectrum that shows both Type A and B metabolite peaks. The peak around 0.7−1.0 ppm is ${\text{CH}}_{3}$ lipids. The peak around 1.1−1.5 ppm is ${\text{CH}}_{2}$ lipids. The peak around 5.1−5.5 ppm is CH=CH lipids. The horizontal axis represents the chemical shifts of molecules in ppm, and the vertical axis is signal amplitudes. “Origin” means the original raw spectrum after all spectral corrections (zero filling, baseline correction, phase correction, and apodization) and “estimate” means the fitted spectrum.

In the studied fluids, we found the three typical types of spectra, as shown in Fig. 2. The metabolite assignments were: 1) vinylic CH=CH lipids around 5.1−5.5 ppm, that is, mobile lipids and lipoproteins; 2) ${\text{CH}}_{2}$ lipids around 1.1−1.5 ppm; and 3) ${\text{CH}}_{3}$ lipids around 0.7−1.0 ppm that are bulk ($-{\text{CH}}_{2}$‐)n chains and fatty acyl chain terminal‐end ${\text{CH}}_{3}$ resonances from mobile lipids and lipoproteins. These resonances represent the mobile molecular components of the fatty acids. In a previous *ex vivo* study,^(3)^ the peak intensities of those resonances were very low, but they were very high in the current *in vivo* study. In Fig. 2, the horizontal axis represents the chemical shifts in the molecules in ppm, and the vertical axis is the signal amplitudes. “Origin” means the original raw spectrum after all spectral corrections (zero filling, baseline correction, phase correction, and apodization), and “estimate” means the fitted spectrum.

Table 1 summarizes the means and standard deviations of the metabolites for each disease group. Age was significantly different among all three groups (p=0.0007, Kruskal‐Wallis test), as was gender (p=0.0004, Chi‐squared test). In the post hoc analysis, age was significantly different between the degenerative group and the traumatic as well as infectious groups. There were more females than males in the degenerative group, but more males than females in the traumatic group.

There were no significant differences among the three disease groups for the ${\text{CH}}_{3}$ (p=0.9019), ${\text{CH}}_{2}$ (p=0.6406), and CH=CH (p=0.5467) lipids and water (p=0.2853). In addition, there were also no significant differences among the three groups in the sum of the ${\text{CH}}_{3}$ and ${\text{CH}}_{2}$ lipids (p=0.6886), in the ratio of the ${\text{CH}}_{3}$ to ${\text{CH}}_{2}$ lipids (p=0.1700), or in the ratio of the CH=CH lipids to the sum of the ${\text{CH}}_{2}$ and ${\text{CH}}_{3}$ lipids (p=0.3696) and LI (p=0.3696). The intensities of the ${\text{CH}}_{2}$ lipid (mean±standard error, 0.0125±0.00279) for the 84 patients were usually greater than those of the ${\text{CH}}_{3}$ lipid (mean±standard error, 0.0019±0.0004) (p<0.0001, Wilcoxon test). The resonance intensity ratios of the ${\text{CH}}_{3}$ to ${\text{CH}}_{2}$ lipids were therefore substantially smaller than 1.0 for most patients. Forty‐one patients out of 84 (48.8%) were categorized into the Type A spectrum with the range of LI=0.5 to 1.0. Nine patients (10.7%) were in the Type B spectrum with the range of LI=−0.5 to −1.0, which means that no metabolic peaks were found in the CH=CH signal intensities in these nine patients. Thirty‐four of the 84 (40.5%) patients were in the Type C spectrum with the range of LI=−0.5 to 0.5. Among all 84 patients, the metabolites for the ${\text{CH}}_{2},{\text{CH}}_{3}$, and CH=CH lipids and the sum of the ${\text{CH}}_{2}$ and ${\text{CH}}_{3}$ lipids as well as water were not significantly different between males and females (p>0.1202).

As listed in Table 1, in the degenerative disease group (total 21), 13 spectra were Type A (61.9%), 5 were Type B (23.8%), and 3 were Type C (14.3%). In the traumatic disease group (total 12), 7 spectra were Type A (58.3%), 3 were Type B (25.0%), and 2 were Type C (16.7%). In the infectious and inflammatory disease group, all spectra were Type A (100%). The spectrum types were not significantly different among the three groups (p=0.550, Chi‐squared test); both the degenerative and traumatic disease groups had all three types, but Type A was dominant in both groups. However, the infectious disease group had only the Type A spectrum. Among 38 patients, 25 were in the Type A spectrum group (65.8%), 8 were in the Type B group (21.1%), and 5 were in the Type C group (13.2%).

**Figure 3:**
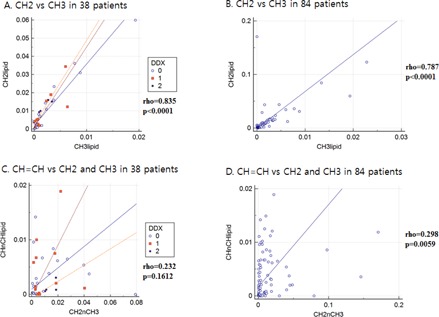
Results of Spearman's rank correlation tests. The differential diagnosis (DDX) indicates 0 for degenerative, 1 for traumatic, and 2 for infectious and inflammatory disease groups. ${\text{CH}}_{2}$ lipids in the 38 patients for whom we had confirmed diagnoses (3A, rho=0.835, p<0.0001) and for all 84 patients including those with unknown diagnoses (3B, rho=0.787, p<0.0001) were significantly correlated with ${\text{CH}}_{3}$ lipid. CH=CH lipid (CHnCHlipid) in the 38 patients (3C, rho=0.232, p=0.1612) was not significantly correlated with the sum of the ${\text{CH}}_{2}$ and ${\text{CH}}_{3}$ lipids (${\text{CH}}_{2}$n${\text{CH}}_{3}$), but for all 84 patients (3D, rho=0.298, p=0.0059), the correlation was significant.

Figure 3 shows the results of the Spearman rank correlation tests. The ${\text{CH}}_{2}$ lipids for the 38 patients whose diagnoses were confirmed (3A, rho=0.835, p<0.0001) and for all 84 patients including those with unknown diagnoses (3B, rho=0.787, p<0.0001) were significantly correlated with ${\text{CH}}_{3}$ lipids. In addition, the CH=CH lipid (CHnCHlipid) in the 38 patients with known diagnoses (3C, rho=0.232, p=0.1612) was not significantly correlated with the sum of the ${\text{CH}}_{2}$ and ${\text{CH}}_{3}$ lipids (${\text{CH}}_{2}$n${\text{CH}}_{3}$), although there was a significant correlation among all 84 patients including those with unknown diagnoses (3D, rho=0.298, p=0.0059). Age in the full group of 84 patients was not correlated with any of the metabolites in any of the lipids (CH=CH: rho=0.187, p=0.088; CH2: rho=−0.036, p=0.7428; CH3: rho=−0.037, p=0.7366; sum of ${\text{CH}}_{2}$ and ${\text{CH}}_{3}$: rho=−0.0305, p=0.7833) or with water (rho=−0.0406, p=0.7141).

**Figure 4:**
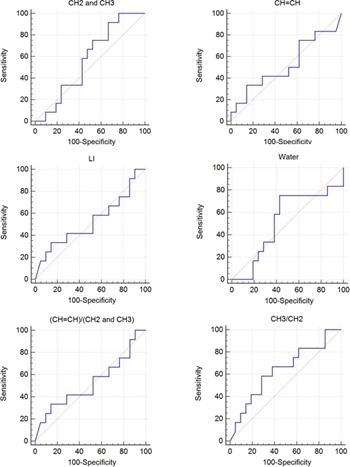
Plots of the receiver operating characteristic (ROC) curve analysis results between the degenerative and traumatic disease groups. Table 2 lists area under the ROC curve (AUC) values, 95% confidence interval (95% CI), Z‐statistic (*p*, p‐value), sensitivity (%), and specificity (%). None of the metabolites could distinguish between the two diseases (p>0.192).

ROC curve analyses were performed for the degenerative and traumatic disease groups using multiple metabolites (Table 2). For the sum of ${\text{CH}}_{2}$ and ${\text{CH}}_{3}$ lipids, sensitivity was good, but specificity was poor. For the ratio of the CH=CH lipid to the sums of the ${\text{CH}}_{2}$ and ${\text{CH}}_{3}$ and the ${\text{CH}}_{3}$ to ${\text{CH}}_{2}$ lipids, sensitivity was poor, but specificity was good. None of the metabolites could differentiate between the two diseases (p>0.192). Figure 4 shows the plots of the ROC curve analyses.

**Table 2:** Results of the receiver operating characteristic (ROC) curve analyses of metabolites between the degenerative and traumatic disease groups

*Metabolites*	*AUC*	*95% CI*	*Z (p)*	*Sensitivity (%)*	*Specificity (%)*
${\text{CH}}_{2}$ and ${\text{CH}}_{3}$	0.571	0.388 to 0.742	0.706 (0.480)	91.67	33.33
CH=CH	0.524	0.343 to 0.700	0.208 (0.8350)	33.33	85.71
LI	0.528	0.347 to 0.703	0.244 (0.8074)	33.33	85.71
Water	0.520	0.340 to 0.696	0.178 (0.8591)	75.00	57.14
Ratio $\left(\text{CH}=\text{CH})/({\text{CH}}_{2}\;\text{and}\;{\text{CH}}_{3}\right)$	0.528	0.347 to 0.703	0.244 (0.8074)	33.33	85.71
Ratio ${\text{CH}}_{3}/{\text{CH}}_{2}$	0.637	0.452 to 0.796	1.305 (0.1920)	58.33	71.43

LI=lateralization index, calculated as LI=(PA−PB)/(PA+PB), where PA was the sum of the peak intensity of ${\text{CH}}_{2}$ and ${\text{CH}}_{3}$ and PB was the sum of CH=CH; Water=water reference peak; AUC=area under the ROC curve; CI=confidence interval; Z=Z‐statistic; p=p‐value.

## IV. DISCUSSION

The goal of this study was to investigate the usefulness of proton MRS to differentiate between degenerative, traumatic, and infectious diseases by evaluating metabolites in the joint fluid regions in human knees using a 3T MR system.

### A. Three types of spectra were found

The visible peaks in the spectra of the joint fluid areas in knees *in vivo* were assigned to one of three lipid signals: 1) ${\text{CH}}_{3}$ lipid protons around 0.9 ppm (i.e., 0.7−1.0 ppm), 2) ${\text{CH}}_{2}$ lipid protons around 1.3 ppm (i.e., 1.1−1.5 ppm), and 3) CH=CH lipid with alpha glycine protons around 5.4 ppm (i.e., 5.1−5.5 ppm). Each patient had a CH=CH lipid peak, ${\text{CH}}_{2}$ and ${\text{CH}}_{3}$ peaks, or both CH=CH and ${\text{CH}}_{2}$ and ${\text{CH}}_{3}$ peaks. The ${\text{CH}}_{3}$ lipid protons are methyl protons. The ${\text{CH}}_{2}$ lipid protons around 1.3 ppm are aliphatic chain methylene protons, separated by resonance peaks around 1.5 ppm from the CH protons of extracellular necrotic lipids and macromolecules, and it is supposed that the shifted resonance at about 1.3 ppm could be attributed to intracellular lipids in joint effusion that experience different bulk susceptibility.^(27)^

In the degenerative disease group, we found a large peak around 1.5 ppm, which is ${\text{CH}}_{2}$ and ${\text{CH}}_{3}$ lipids with or without minor metabolites around 5.4 ppm (Type A spectrum). Most of the patients (61.9%) were in this spectrum type, followed by patients (23.8%) who had a large peak around 5.4 ppm with or without minor metabolites around 1.5 ppm, which is CH=CH lipid (Type B spectrum). Large peaks were found in 14.3% of patients, at 1.5 ppm and 5.4 ppm (Type C spectrum). In the traumatic disease group, 58.3% of patients had ${\text{CH}}_{2}$ and ${\text{CH}}_{3}$ lipid peaks, and we also found all three types of spectra in this group. In the infectious and inflammatory disease group, all five patients had ${\text{CH}}_{2}$ and ${\text{CH}}_{3}$ lipid peaks. We did not find any large peaks at 5.5 ppm.

### B. Metabolites in the effusion region cannot differentiate between diseases

None of the lipids, ${\text{CH}}_{2},{\text{CH}}_{3}$, or CH=CH, could significantly differentiate between the degenerative, traumatic, and infectious diseases (Tables 1 and 2). This result was unchanged when the metabolite was normalized by the intensity of water. Calculating the lateralization indexes of the metabolite peaks did not help to differentiate among the three groups, and nor did mathematical manipulation of the metabolites. In this study, the sensitivity and specificity of the ROC curve analyses were low (Table 2). Therefore, rather than differentiating among the three groups using the metabolite peaks, it may be important to investigate why a patient has ${\text{CH}}_{2}$ and ${\text{CH}}_{3}$ lipids but minimal CH=CH lipid or vice versa, as well as why a patient has both ${\text{CH}}_{2}$ and ${\text{CH}}_{3}$ lipids and CH=CH lipid. This investigation may give us clues about disease mechanisms. Although the metabolites were not significantly different among the three groups (Table 2), the infectious disease group had a large ${\text{CH}}_{2}$ and ${\text{CH}}_{3}$ lipid peak with a low CH=CH lipid peak. This may be a hint at the different mechanisms between infectious, degenerative, and traumatic diseases. The results of previous ex vivo NMR spectroscopy studies^(18)^ showed that metabolic signals of normal synovial fluid samples were different from those of inflammatory fluid samples. The spectra of synovial fluid samples on patients with inflammatory joint diseases contained higher levels of lipoprotein‐associated fatty acids than did those for normal synovial fluid samples.^(18)^ Because of the very small number of patients in the infectious disease group in this study, further research is required with a large population to evaluate this finding. Although understanding the mechanisms is beyond the scope of this study, we thought that the ${\text{CH}}_{2}$ and ${\text{CH}}_{3}$ lipid peaks could be produced by intramyocellular damages caused by infectious, degenerative, or traumatic diseases, but the CH=CH peak of unsaturated acid lipid protons can be generated by muscular damages caused by degeneration or traumatic events. However, to provide insight into the underlying proton metabolism in joint fluids, future studies could investigate quantifying metabolites to identify acute and chronic states of effusion causes.

The mechanisms for why different patterns of lipid peaks are created were unclear in the current study, but it could be the outcome of variable joint fluid production in different conditions. One of the major metabolites in *in vivo* proton MR spectroscopy of muscles is lipids, which are major substrates for energy production both at rest and during muscle contraction.^(28)^ A previous study found that large variations in low‐molecular‐weight species in *ex vivo* synovial fluid samples were found between individuals and that there was no measurable correlation between disease state and the levels of any low‐molecular‐weight components.^(22)^ This ex vivo study found higher levels of triglycerides, namely, from the ${\text{CH}}_{3},{\text{CH}}_{2}$, and vinylic CH groups, as well as more creatine in patients with traumatic effusions than in those with rheumatoid arthritis or osteoarthritis.^(22)^ However, we did not find this difference. Although our study did not show any significant differentiation among the three patient groups, our study may guide future works for investigating *in vivo* MR spectroscopy in the knee.

### C. The ratio of ${\text{CH}}_{3}$ to ${\text{CH}}_{2}$ was highest in the degenerative disease group

Although the ratio of ${\text{CH}}_{3}$ to ${\text{CH}}_{2}$ did not show any significant differences among the groups, it was less than 1 in all patients. Furthermore, the ratio was highest in the degenerative disease and lowest in the infectious disease (Table 1) groups. The ratio was 1.9 times greater in the degenerative disease group and 1.4 times greater in the traumatic disease group than in the infectious disease group. With more patients, this ratio may be useful for distinguishing patient groups. A previous study showed that the ratio of ${\text{CH}}_{3}$ to ${\text{CH}}_{2}$ intensity gives an approximate measure of the average chain length of glycerides, and the intensity of the CH=CH signal gives a measure of the amount of unsaturated fatty acid present.^(22)^ The larger ratio of ${\text{CH}}_{3}$ to ${\text{CH}}_{2}$ may indicate osteoarthritis with a shortened apparent chain length and reduced amounts of saturated fatty acids. In this present study, intensities of ${\text{CH}}_{2}$ lipids were always higher than those of ${\text{CH}}_{3}$ lipids in all patients. The ratio of CH=CH intensity to the sum of the ${\text{CH}}_{2}$ and ${\text{CH}}_{3}$ lipids was higher in both the degenerative (0.992) and the traumatic (0.915) disease groups than the infectious disease group (0.143). This ratio may also be used to distinguish patients with degenerative and traumatic diseases from those with infectious diseases. However, further study is necessary.

Lower ${\text{CH}}_{3}$ levels corresponded to lower ${\text{CH}}_{2}$ levels. The results of the previous *ex vivo* NMR study^(22)^ showed that the ratio of ${\text{CH}}_{3}$ to ${\text{CH}}_{2}$ was higher in osteoarthritis than in rheumatoid arthritis or in traumatic fluid. Low levels of ${\text{CH}}_{2}$ and ${\text{CH}}_{3}$ were indicated by marked reductions in the amounts of saturated fatty acid present. The level of triglyceride of ${\text{CH}}_{2}$ was relatively decreased compared with that of ${\text{CH}}_{3}$ in patients with osteoarthritis and rheumatoid arthritis. The highly localized concentrations of free fatty acids resulted in toxic effects. In the previous study, the signal intensities for the ${\text{CH}}_{3}$ or ${\text{CH}}_{2}$ and CH protons of triglycerides did correlate with each other,^(22)^ but there was no correlation in this study.

### D. Study limitations

This study had some limitations. First, although we studied 84 patients, only 38 had confirmed differential diagnoses. Furthermore, the number of patients in the infectious and inflammatory diseases group was too small and, as such, additional studies with large numbers of patients should be performed. Second, MR spectroscopy should be performed with and without suppressing both water and lipid peaks to locate and quantify small amounts of metabolisms *in vivo* that were not shown in this study. Strong lipid signals can hinder accurate estimations of weak metabolite signals. A previous *ex vivo* study also showed large variations in the low‐molecular‐weight species in synovial fluid samples between individuals, but there was no measurable correlation between disease states and levels of any low‐molecular‐weight components.^(22)^ This study did not focus on identifying signals from low‐molecular‐weight metabolites. Third, MRS should be also performed with two different echo times to distinguish lipid signals from lactate signals. A previous *ex vivo* animal study showed that lactate was different in osteoarthritic fluids compared with normal synovial fluids.^(24)^ Finally, the effusion samples used to acquire the MR spectrum should be extracted to evaluate sample components using a chemical assay method to verify the compounds in the spectrum. MRS is a sensitive probe of local molecular environments. Thus, changes in MRS spectral peak intensity or line width can correlate directly with variations in metabolic species or environmental conditions. Identifying these changes can provide important insights into the biomolecular mechanisms that underlie disease processes as well as providing treatment responses. Because chemical shift resolution is doubled at 3T compared with 1.5T, spectral resolution is expected to be improved. In this study, we used short echo time (TE) to improve signal‐to‐noise ratio and to identify any unknown components in effusion areas that may disappear at long TEs. However, an intermediate TE may be applicable for determining differences between lactate and lipid signals.

## V. CONCLUSIONS

This study reported the characteristics in effusion areas in the knee using *in vivo* single‐voxel spectroscopy and a 3T MRI system in different types of diseases. In both degenerative and traumatic diseases, metabolite peaks of the vinylic CH=CH lipid around 5.1−5.5 ppm and of the sum of the ${\text{CH}}_{2}$ and ${\text{CH}}_{3}$ lipids around 0.7−1.5 ppm were observed, but in the infectious disease, the only metabolite peak detected was in the sum of the ${\text{CH}}_{2}$ and ${\text{CH}}_{3}$ lipids. The ratio of ${\text{CH}}_{3}$ to ${\text{CH}}_{2}$ was highest in the degenerative diseases. To the best of our knowledge, this study was the first report of in vivo MR spectroscopy of the joint fluid area in human knees using a 3T MRI system. Although none of the metabolites could statistically significantly differentiate between the three types of diseases, the different lipid metabolite peaks in the three disease groups may be a hint at the different mechanisms between infectious, degenerative, and traumatic diseases. Additional studies must be performed with more patients.

## ACKNOWLEDGMENTS

This work was supported by a grant from the Kyung Hee University in 2012 (KHU‐20120762).

## COPYRIGHT

This work is licensed under a Creative Commons Attribution 4.0 International License.
